# Multicenter Clinical Management Practice to Optimize Outcomes Following Tendyne Transcatheter Mitral Valve Replacement

**DOI:** 10.1016/j.shj.2022.100025

**Published:** 2022-04-04

**Authors:** Alison Duncan, Gry Dahle, Lenard Conradi, Nicholas Dumonteil, John Wang, Nimesh Shah, Benjamin Sun, Paul Sorajja, Gorav Ailawadi, Jason H. Rogers, Cesare Quarto, Brian Bethea

**Affiliations:** a Heart Division, The Royal Brompton Hospital, London, UK; b Department of Cardiothoracic Surgery, Oslo University Hospital, Oslo, Norway; c Department of Cardiothoracic Surgery, University Heart and Vascular Center Hamburg, Hamburg, Germany; d Department of Cardiovascular Medicine, Groupe CardioVasculaire Interventionnel, Clinique Pasteur, Toulouse, France; e Cardiovascular Intensive Care Unit, MedStar Union Memorial Hospital, Baltimore, Maryland, USA; f Department of Cardiothoracic Surgery, Minneapolis Heart Institute, Minneapolis, Minnesota, USA; g Center for Valve and Structural Heart Disease, Minneapolis Heart Institute, Minneapolis, Minnesota, USA; h Department of Cardiothoracic Surgery, University of Michigan, Ann Arbor, Michigan, USA; i Department of Cardiovascular Medicine, Davis Medical Center, Sacramento, California, USA; j Department of Cardiothoracic Surgery, The Royal Brompton Hospital, London, UK; k Department of Cardiothoracic Surgery, MedStar Union Memorial Hospital, Baltimore, Maryland, USA

## Introduction

**Figure 1:**
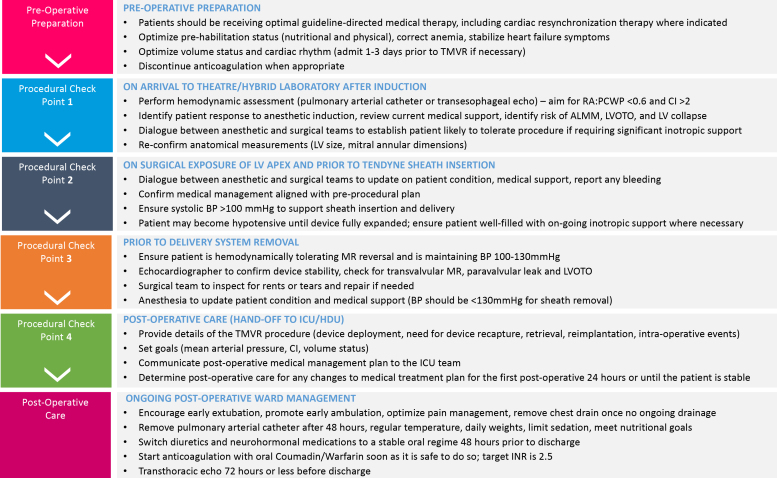
**Main recommendations for before, during, and after Tendyne-TMVR.** Abbreviation: TMVR, transcatheter mitral valve replacement.

The Tendyne^TM^ transcatheter mitral valve replacement system (Tendyne-TMVR, Abbott Structural Heart, Santa Clara, California) is a therapy for patients with symptomatic mitral valve (MV) disease high risk for conventional MV surgery or challenging anatomy for transcatheter MV repair. With over 1000 Tendyne-TMVR performed worldwide and beneficial periprocedural, 30-day, and 2-year outcomes,^1^ the aim of this article is to present best practice recommendations from expert centers performing Tendyne-TMVR to support optimal outcomes (Figure 1).

## Preoperative Preparation

Patients undergoing Tendyne-TMVR should be receiving optimal tolerated guideline-directed medical therapy according to American College of Cardiology Foundation/American Heart Association Heart Failure Guidelines^2^ prior to intervention. Prehabilitation (nutritional/physical) should be optimized including correction of iron deficiency anemia. Patients with heart failure or cardiac-related hospitalizations should have stable volume status prior to intervention, and the procedure should not be considered for emergent or urgent situations. All patients should be discussed in multidisciplinary team format composed of interventional cardiologists, surgeons, imaging specialists, and heart failure cardiologists; additional team members include cardiac anesthesia and cardiac critical care members, and geriatrician assessment is useful to quantify patient frailty. In-hospital heart failure optimization for 1-3 days prior to Tendyne-TMVR should be considered for patients who require volume status management to avoid significant periprocedural volume shifts. Cardiac rhythm should be stable and electrophysiological optimization considered if there is evidence of new rhythm changes. Permanent pacing systems should be programmed to avoid asynchronous pacing. Anticoagulation should be discontinued according to published guidelines.^3^

## Preprocedural Risk Assessment

Preprocedural right heart catheterization should be considered in all patients, particularly those with severe pulmonary hypertension (pulmonary artery systolic pressure [PASP] ≥60 mmHg on echo), moderate/severe right ventricular (RV) dysfunction (tricuspid annular plane systolic excursion <15 mm, S’ <10 cm/s, fractional area change <30%), and/or signs of right heart congestion (moderate tricuspid regurgitation, worsening renal/hepatic function). Revascularizing severe coronary disease is recommended. Stress echocardiography should be considered in patients with left ventricular (LV) ejection fraction <30% to assess the presence of contractile reserve.

Potential periprocedural complications during Tendyne-TMVR include afterload mismatch (ALMM), LV outflow tract (LVOT) obstruction (LVOTO), and LV collapse. ALMM may occur when acute elimination of MR causes a sudden reduction in LV and/or RV systolic function that is poorly tolerated. The combination of ALMM and LVOTO can be particularly challenging to manage, so it is imperative to identify these patients preprocedurally.

### Patients at Risk of ALMM

With LV dysfunction are patients with LV ejection fraction <40%, dilated LV (end-diastolic dimension >6 cm, end-systolic dimension >4 cm), cardiac output < 3.0 L/min, or cardiac index (CI) < 2.0. Patients at risk of ALMM and biventricular dysfunction are those with RV dysfunction and/or PASP ≥60 mmHg.

### Patients at Risk of LVOTO

These include patients with preprocedural predicted neo-LVOT values in the range of 250 mm^2^ or less, small LV (end-systolic dimension <3.5 cm), dynamic basal septum, long (>28 mm) unsupported anterior mitral valve leaflet (AMVL), narrow (<6 mm) distance between AMVL and septal edge in systole, narrow (<120°) aortomitral angle, and anterior apical access point (intentional or unintentional).^4^

### Patients at Risk of LV Collapse

These include those with small left ventricle (<3.5 cm), basal septal hypertrophy, or ischemia.

## Intraprocedural Management

Frequent communication between Tendyne-TMVR implant and anesthetic teams is vital to minimize periprocedural and postprocedural complications. Surgical, anesthetic, and imaging teams should participate in the team brief to ensure the entire periprocedural team is aware of individualized patient hemodynamic risks, medical management plan, and surgical bailout options. It is important to communicate that the final delivery phase is often associated with hypotension and to discuss in advance the balance between patience vs. immediate corrective actions. Following the team brief, there are 4 “checkpoints” when the patient’s condition should be re-reviewed, paying particular attention to the patient’s hemodynamic data, volume status, laboratory data (coagulation, arterial blood gases), changes in medications, and re-review of the medical management plan.

### Checkpoint #1: On Arrival to Theater/Hybrid Laboratory After Induction

Continuous cardiac monitoring throughout the periprocedural period is recommended. A pulmonary arterial catheter permits estimation of right atrial pressure, PASP, cardiac output, and CI. Optimal preprocedural hemodynamics include right atrial pressure-to-pulmonary capillary wedge pressure ratio <0.6 and CI >2. Pigtail catheter insertion in the left ventricle is helpful to assess LVOT pull-back gradient. Transesophageal echocardiography (TEE) should confirm anatomical measurements (LV size, mitral valve annular dimensions) to ensure no changes from screening. Temporary pacing should be considered in patients with no permanent system who are at risk of ALMM or LV collapse in whom programming heart rate at 80-100 bpm may compensate for a relatively low stroke volume. The implant team should ascertain the patient’s response to induction of anesthesia, identify hemodynamic changes from baseline, acknowledge current medical support, and re-establish whether the patient is likely to tolerate the procedure if requiring significant levels of inotropic support.

### Checkpoint #2: On Surgical Exposure of the LV Apex and Prior to Tendyne-TMVR Sheath Insertion

Surface transthoracic echo (TTE) identifies the optimal intercostal space for LV apical access identified on preprocedural multislice computerized tomography. Once confirmed, the surgical team may extend the parietal pleural and intercostal muscles to improve apical exposure and minimize rib fracture risk. An Alexis soft-tissue retractor (Applied Medical, CA) and mini thoracotomy retractor are used, but it may be necessary to surgically divide a rib in patients with reduced apical visualization. Surgical preparation of the LV apex for apical puncture may be performed using 3-0 SH-1 pledgeted sutures in a U-stitch, Star of David, or classic purse string formation. After optimizing TEE X-plane LV and MV imaging, the echocardiographer should confirm LV apical access point during simultaneous LV apical finger palpation by the surgical implanter (after each palpation, the LV apex should be visually assessed to exclude hematoma). Before transapical puncture, the implant team should communicate with the anesthetist for an update on the patient’s condition and medical support and ensure systolic blood pressure is > 100 mmHg to support device insertion and delivery.

## Intraoperative Care Pathways

Following placement of LV apical pledgeted sutures and administration of heparin to achieve an activated clotting time >300 seconds, a 0.035-inch guidewire is inserted into the left atrium (LA) via the LV, and a Fogarty balloon advanced/retracted over the wire to ensure no entanglement with the mitral subvalvular apparatus. A 34Fr delivery sheath is inserted into the LA using an over-the-wire technique, and the Tendyne-device is introduced through the sheath, partially unsheathed in the LA, aligned with the aortomitral continuity using TEE, and retracted until the device cuff rests on the LA floor. The remainder (80%) of the prosthesis is deployed within the annulus and secured with a braided, high-molecular-weight polyethylene tether attached to an epicardial pad. The patient may become hypotensive until the device is fully expanded, so the patient should be well filled with inotropic support if necessary (dopamine, dobutamine, or milrinone), noting transient low output usually resolves on complete device deployment and so excessive use of inotropes (LVOTO risk) should be avoided. The surgeon should not lower the delivery sheath trajectory as this results in systolic anterior motion of the AMVL and LVOTO. At the procedure end, the tether tension is adjusted to optimize seating of the prosthesis. The implant team (surgical, anesthetic, imaging) should be alert to potential ALMM, LVOTO, and LV collapse throughout TMVR deployment and complications managed accordingly.

### Patients at Risk of ALMM

**Figure 2:**
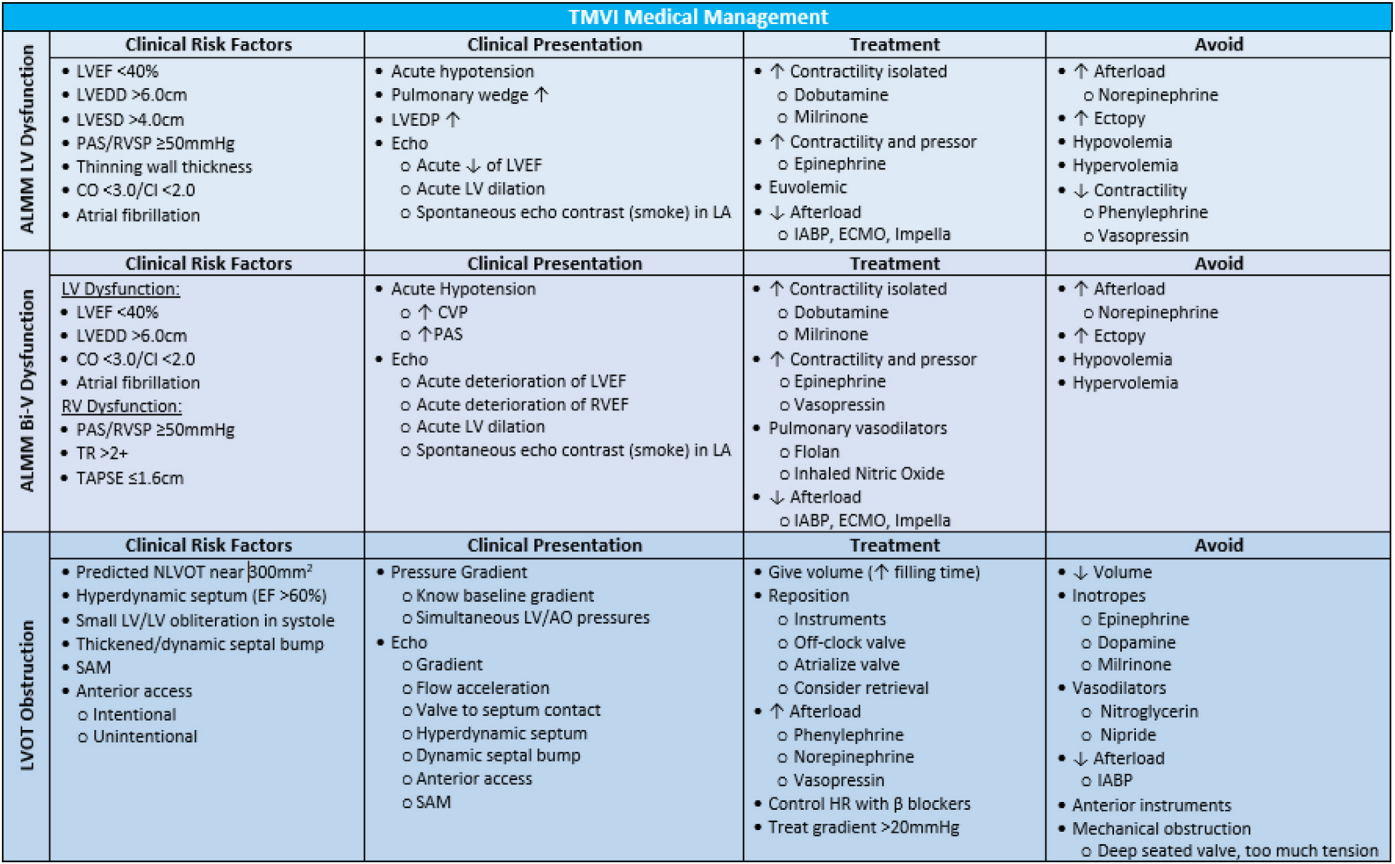
**Proposed clinical management of potential complications during Tendyne-TMVR.** Abbreviation: TMVR, transcatheter mitral valve replacement.

Care should be exercised to prevent hypotension caused by AMVL distortion. Inotropic support should be considered with a low threshold for reducing afterload with mechanical support (intra-aortic balloon pump, Impella) or extracorporeal mechanical oxygenation for extreme hemodynamic collapse (Figure 2). Pulmonary vasodilators must be considered if RV dysfunction is significant. Zealous use of peripheral vasoconstrictors, hyper/hypovolemia, and significant increase in afterload should be avoided.

### Patients at Risk of LVOTO

Volume resuscitation, increasing afterload, and beta-blockade must be considered to avoid tachycardia. Inotropic and chronotropic medications must be avoided when possible, and if hemodynamic instability persists, device repositioning, lateral device rotation to optimize LVOT opening (“off-clocking”), atrialization, or device retrieval must be considered.

### Complete LV Collapse

Volume resuscitation and inotropic support will be required with backup pacing (epicardial if urgent, transvenous via temporary wire), or mechanical support.

### Checkpoint #3: Prior to Delivery System Removal

It is important to ensure from the anesthetist that the patient is tolerating MR reversal and maintaining adequate systolic blood pressure; from the echocardiographer that the Tendyne device is stable and that transvalvular MR, paravalvular leak, and LVOTO have been excluded; and from the surgical team that there is adequate apical hemostasis. The patient’s blood pressure should be < 130 mmHg to facilitate sheath removal. If the patient is stable, the surgical team should proceed with device decoupling and apical pad placement. Apical bleeding should be repaired before delivery sheath removal (a short period of cardiopulmonary bypass may be required).

## Immediate Postprocedural Management

### Checkpoint #4: Postoperative Care

Postoperative management is key to successful outcomes, and patients should be managed like cardiac surgical patients. A clear and algorithmic handover to intensive care/high-dependency unit/ward is vital for the safe progression of the patient from theater/hybrid lab to hospital discharge. The implant team should detail the Tendyne-TMVR procedure, set hemodynamic goals (mean arterial pressure, CI, volume status), and communicate the postoperative medical management plan. A decline in cardiac performance can be subtle in the immediate postoperative period, and vigilance is required. The implant team should be informed of any bleeding or changes in cardiac hemodynamics, and large shifts in the patient’s volume status (including overdiuresis) should be avoided. Early extubation ambulation should be encouraged. Pain management is critical, and intercostal or paravertebral nerve block should be considered with removal of chest tubes once there is no evidence of ongoing drainage. Left pleural effusion is common and may cause delayed respiratory insufficiency; drainage may be required. Pulmonary arterial catheter and arterial lines may be removed after 48 ​hours, and serial echocardiography used to determine hemodynamics. Close attention should be paid to rhythm changes, and new-onset atrial fibrillation cardioverted expeditiously to avoid decompensation. Pacing may be used to optimize heart rate in patients at risk for ALMM. On transfer to ward-based care, patients should be encouraged to mobilize, sedation medication limited, sleep/wake cycles optimized, and nutritional goals met. Diuretics and neurohormonal medications should be switched to a stable oral regime 48 ​hours prior to discharge and beta-blockers titrated cautiously. TTE must be performed 72 ​hours or less before discharge to document LV/RV size and function, mitral valve and LVOT gradients, and any paravalvular leak. Anticoagulation with oral Coumadin/Warfarin may be started as soon as safe (often the first postoperative day) to a target international normalized ratio (INR) 2.5 (discontinue bridging subcutaneous low-molecular-weight heparin when INR ≥2.0). Anticoagulation should be for at least 6 months and lifelong if well tolerated. Antiplatelet therapy should be continued as per standard recommendations. Novel oral anticoagulants are not currently recommended, though this is subject to ongoing review in selected cases.^5^

## Follow-up After Discharge

Recommended clinical review with INR check and TTE should be performed 4 weeks postprocedure. Patients should be counseled to contact the medical team if there is any wound infection, pain, breathlessness, palpitations, dizzy spells, and fatigue not resolving with rest between discharge and planned clinical review.

## Conclusion

We present current best practice recommendations on periprocedural management for Tendyne-TMVR, based on consensus from a relatively small number of experienced centers worldwide. These recommendations may change with technological iterations, increasing clinical experience, and expanded evidence base.

## Funding

The authors have no funding to report.

## Disclosure statement

Alison Duncan receives consultancy fees from Abbott Vascular, Edwards Lifesciences, Medtronic, and NeoChord. Gry Dahle receives consultancy fees from Abbott Vascular. Lenard Conradi receives consultancy fees from Abbott Vascular. Nicholas Dumonteil receives consultancy fees from Abbott Vascular, Ancora Heart, Boston Scientific, Edwards Lifesciences, and Medtronic. Nimesh Shah receives consultancy fees from Edwards Lifesciences. Benjamin Sun receives proctoring and consultancy fees from Abbott Vascular. Paul Sorajja receives consultancy fees from Anteris, Abbott Structural, Medtronic, Boston Scientific, WL Gore, TriFlo, and Vdyne. Gorav Ailawadi receives consultancy fees for Abbott, Edwards, Medtronic, Admedus, Gore, and AtriCure. Jason H Rogers receives research funding from Abbott and Boston Scientific and consultancy fees from Abbott, Boston Scientific, and Gore. Brian Bethea receives consultancy fees from Abbott Vascular. John Wang had no conflict to declare.
